# Poorer Search Efficiency in Healthy Young Adults With High Schizotypal Personality Traits

**DOI:** 10.3389/fpsyt.2018.00285

**Published:** 2018-07-12

**Authors:** Kirsten R. Panton, Johanna C. Badcock, J. Edwin Dickinson, David R. Badcock

**Affiliations:** ^1^Human Vision Laboratory, School of Psychological Science, University of Western Australia, Perth, WA, Australia; ^2^Division of Psychiatry, Faculty of Health and Medical Sciences, Centre for Clinical Research in Neuropsychiatry, University of Western Australia, Perth, WA, Australia; ^3^Cooperative Research Centre - Mental Health, Carlton, VIC, Australia

**Keywords:** schizotypy, perceptual organization, global processing, local processing, schizophrenia

## Abstract

Perceptual organization (PO) difficulties have repeatedly been reported in people with schizophrenia, and in healthy individuals with high levels of schizotypy traits, who are at increased risk for schizophrenia. In particular, poor performance on the Embedded Figures Test (EFT) has been interpreted as an atypically strong preference for global over local processing, even though these processes cannot be clearly disambiguated on this test. Here we use two separate versions of the Radial Frequency Search Task (RFST), a new index of PO abilities, to selectively investigate global and local processing of shape information in trait schizotypy. Schizotypy traits were assessed using the Wisconsin Schizotypy Scales-Brief. Individuals selected for high and low levels of positive schizotypy [assessed with the Wisconsin Schizotypy Scales-Brief Perceptual Aberration (PAb) scale] completed the EFT, along with the Global RFST and the Local RFST, all of which require participants to find a target shape amongst distractor elements. The High PAb group (*n* = 83) were less efficient (i.e., reactions times slowed more as the set size increased) than the Low PAb group (*n* = 146) on the Global RFST (significant group differences for Target Absent conditions only), but not the Local RFST. High and Low PAb groups also differed on other schizotypy traits, so the specificity of the results to positive schizotypy cannot be assured. Unexpectedly, no group differences were observed on the EFT; however, there was a small, but significant, positive correlation between RFST search efficiency and EFT performance, indicating that they shared some common processes. Overall, the pattern of results suggests that global (but not local) processing difficulties may be contributing to the poorer perceptual organization observed in groups with high levels of schizotypy traits. In addition, the confinement of this result to the Target Absent condition suggests that the underlying mechanism involves differences in decisional processes on the RFST between high and low schizotypy groups. The RFST shows promise as a useful tool for measuring specific perceptual organization abilities in non-clinical, and potentially clinical, samples.

## Introduction

Recently there has been a renewal of interest in problems with perceptual organization (PO) experienced by people with, or at increased risk (e.g., schizotypy), for schizophrenia (1–4). PO involves a variety of processes that aid in structuring visual information into coherent patterns, such as contours, groups and objects (5, 6). It has been suggested that these impairments may be specific to psychosis (3, 4, 7, 8). The debated developmental exception to this proposal is the atypical processing abilities reported in individuals with autistic-like traits (9–13).

It has been proposed that PO deficits in schizophrenia are most consistently present when the stimuli used are spatially separated, such as in commonly used contour integration paradigms [eg., the Jittered Orientation Visual Integration task; (14–19)]. There is research to suggest that this impairment may already be present in first-episode patients (15), and tends to worsen as the illness progresses (19). Such findings are consistent with the view that schizophrenia is a neurodevelopmental illness (20–22) whose origins arise long before the onset of psychotic symptoms; though, fewer studies have examined PO in healthy “at-risk” groups.

The Embedded Figures Test (EFT), a traditional, though somewhat different, measure of PO, has revealed a common impairment in schizophrenia and high-risk groups (2), including people with high levels of trait schizotypy who are known to be at increased risk for psychosis (23). The EFT assesses the ability to find a *closed-contour* shape hidden amongst a more complex array (24). However, the EFT suffers from a number of weaknesses. In particular, the specific processes driving impaired performance are unclear. For example, poor performance in individuals with positive schizotypal traits (e.g., unusual perceptual experiences) has been interpreted as a global (i.e., whole stimulus), rather than local (i.e., detail), processing preference (25)—though these stimuli are not able to unambiguously distinguish between global and local processes (9–12). Despite the ambiguity regarding the underlying processes of the EFT, de-Wit and Wagemans (1) highlighted how important traditional PO tasks such as the EFT are to better understand individual differences in local and global processing, which has been suggested to be representative of individual differences in higher order cognitive processing styles.

**Figure 1:**
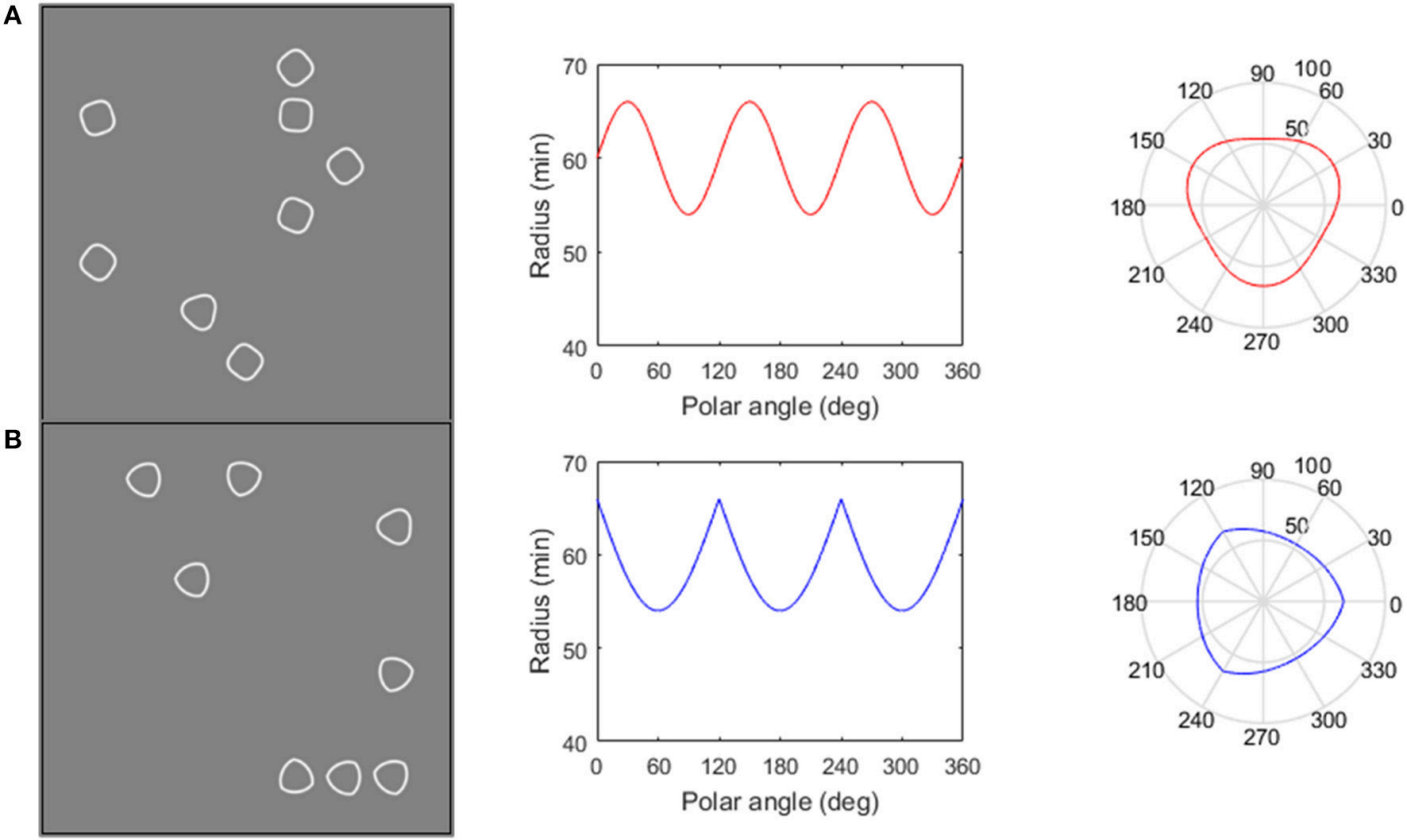
The Global **(A)** and Local **(B)** RFST. Global RFST, **(A)**, column 1: participants must find an RF3 (soft-edged triangle) target amongst RF4 (smooth-cornered squares) distractors, known to be globally processed (47). Local RFST **(B)**, column 1: target and distractors are matched on global descriptors (both RF3s), and are only distinguishable by local differences in curvature or orientation. Column 2 describes how the radii change with variations in polar angle. **(A)** Displays a conventional RF pattern with 3 cycles of modulation (denoted RF3), whereas **(B)** represents a negatively rectified sinusoid. This negative rectification creates curvature discontinuities which manifest as sharp as opposed to smooth corners (see column 3); however, both patterns contain the same number of points of maximum curvature.

In order to overcome the limitations of the EFT, Almeida et al. (9) developed a new measure of one aspect of perceptual organization: the Radial Frequency Search Task (RFST). It uses stimuli known as radial frequency (RF) patterns, which are closed-contour shapes created by deforming a circle, by sinusoidally varying its radius as a function of polar angle [(26); Figure 1]. For this task, individuals are required to identify a target shape [here always a triangle (RF3) with smoothed edges; Figure 1], which is present on 50% of trials, amongst a varied number of distractors. Typically, as the number of distractors present increases (set size = target plus distractors), the time taken to find the target increases. The rate of change in reaction time with increasing set size is referred to as the *slope* of performance, and is used as an indicator of search efficiency. Target Present and Target Absent conditions are also best analyzed separately (9, 27), as they tend to exhibit systematically different response distributions, suggesting that they represent different underlying processes (28). Target Present search conditions are self-terminating (i.e., the participant stops searching when they have found the target), whereas the Target Absent conditions are a serial search task (i.e., the participant searches through the elements until they establish that the target is absent, which optimally requires all elements to be examined).

One advantage of RF patterns is that they allow global and local processes to be clearly separated (11, 28–31). Behavioral studies, for example, have shown that RF patterns with fewer than 10 cycles of deformation (such as RF3 and RF4 stimuli) involve global processing, whilst RF stimuli with more than 10 cycles of deformation engage processes that detect local contour deviations without globally integrating that contour information (28–31). Additionally, imaging and electrophysiological studies implicate area V4 for intermediate shape processing, including globally-processed RF patterns (30, 32). In contrast, prior research, such as that based on the EFT, assumes that global and local processing abilities exist on a continuum. As such, a global processing advantage implies a local processing difficulty (and vice versa), and the two processes cannot be clearly separated (33). In RF pattern research, “local” refers to a local pattern feature (e.g., the orientation difference between a local part of the target contour and a circular contour), and global refers to a property of the contour as a whole, which with these repetitive patterns equates to the angle separating points of maximum curvature (11). Previous studies with the RFST have shown that healthy individuals with high levels of autism-related traits exhibit an advantage on both a Global RFST and a Local RFST (11), signifying not just a local processing advantage, but an overall superiority in this aspect of PO in this group. However, investigations of PO with the RFST in trait schizotypy have not yet been conducted.

The aim of the current study was to examine PO abilities associated with trait schizotypy. Drawing on prior research, we chose to target positive schizotypy traits, specifically high and low scores on the short form of the Perceptual Aberrations (PAb) scale (34). In particular, two previous studies examined PO in high and low positive schizotypy using the EFT, but yielded mixed results, hence conclusions regarding global and local processes remain unclear (25, 35). Furthermore, current explanations of positive schizotypy suggest that the unusual perceptual experiences involved arise from early visual processing difficulties (36). We examined PO ability with two versions of the RFST which specifically target global and local processing, and the EFT. Russell-Smith et al. (25) argued for a global (over local) processing *advantage* in positive schizotypes, therefore, we predicted that individuals with High PAb, compared to Low PAb, scores would exhibit superior search efficiency (shallower search slopes) and faster reaction times (smaller intercepts) on the Global RFST, but not the Local RFST. Drawing on prior literature, we expected reaction times to be longer for the Local RFST, compared to the Global RFST (28), indicating that the former is the more difficult task. We also predicted that the High PAb group would exhibit slower reaction times on the EFT than the Low PAb group (25). To determine the potential specificity of any findings to positive traits, the association with negative and disorganized traits was also explored.

## Methods

### Participants

#### Screening and recruitment

Two thousand six hundred and ninety-three undergraduate students from the University of Western Australia accepted an invitation to complete the Perceptual Aberration (PAb; see Table S1 for further details) and Social Anhedonia scales from the Wisconsin Schizotypy Scales-Brief (37) and the Cognitive Disorganization scale from the short-form Oxford-Liverpool Inventory of Feelings and Experiences (38). Questions were interspersed with items from the Infrequency Scale (39) to identify inconsistent or careless responding. Individuals falling in the 90th percentile (“high” scoring, PAb score ≥ 3) and below the 50th percentile (“low” scoring, PAb score = 0) on the PAb scale were randomly sampled and invited for further testing (outlined below).

#### Clinical assessment and exclusions

The Psychosis Screen (40) provided a brief screen for the presence of psychosis. If 2 (out of 6) items were endorsed, the Diagnostic Interview for Psychosis (41) was used as a more detailed assessment. On clinical interview, participants were excluded if: they self-reported a past or current diagnosis and/or treatment for a psychotic illness (*n* = 2), screened positive for the presence of psychotic illness (*n* = 1), brain injury/disorder (*n* = 3), or substance abuse (*n* = 5) on individual interview. Additional exclusion criteria included poor visual acuity (lower than 20/32, *n* = 0) or poor fluency in English (*n* = 0).

#### Participant characteristics

Ninety-two participants met the inclusion criteria and were recruited in the High PAb group and 170 participants were recruited in the Low PAb group. The sample was predominantly female (68%), young adults (*M*_age_ = 19.39, *SD*_age_ = 2.75, 17–31 years).

### Psychometric measures

#### Schizotypal traits

The Wisconsin Schizotypy Scales-Brief (34) and short-form Oxford-Liverpool Inventory of Feelings and Experiences^1^ (38) self-report scales provide valid and reliable assessments of schizotypy traits (37, 42, 43). *Positive* (Perceptual Aberration (PAb) and Magical Ideation) and *Negative* (Social Anhedonia and Physical Anhedonia) schizotypy were measured using the Wisconsin Schizotypy Scales-Brief (15-items per scale), and *Disorganized* schizotypy was measured with the 11-item Cognitive Disorganization scale of the short-form Oxford-Liverpool Inventory of Feelings and Experiences.

#### Autism-related traits

The Autism-Spectrum Quotient (AQ) was used to measure autistic-like traits in the general population [higher scores = more autistic-like behavior; (44)]. AQ scores were included as a potential confound, given that individuals with high AQ traits (total scores ≥ 23) tend to have improved performance on the RFST (9–12).

### Stimuli and procedure

#### Radial frequency search task (RFST)

The RFST is a computerized visual search task that measures a participant's ability to as quickly and accurately as possible, find a target amongst distractors. The target and distractors were Radial Frequency (RF) patterns (26), which are created by the sinusoidal modulation of the radius of a circle as the function of the polar angle (see Equation 1^2^ By altering parameters of this equation we were able to create shapes with flat sides resembling triangles (RF3s that served as the target shape in both the Global RFST and Local RFST) and squares (RF4s that served as Global RFST distractors)^3^. Furthermore, rectifying the RF stimuli creates discontinuities in orientation (Equation 2^2^), giving the appearance of sharp-cornered triangles (RRF3s, used as Local RFST distractors, since only the corner curvature varies). The number of shapes on the screen (set size) was 1, 2, 4, and 8. The rate of increase in reaction time as set size increased provided a measure of *search efficiency*, our primary measure of performance on this task.

#### Global RFST

The Global RFST involved finding an RF3 (triangle-shape) amongst an array of RF4's (square-shaped), which differ in their global properties (e.g., polar angle between adjacent corners, see column three, Figure 1) and are both known to be globally processed (31, 45–47).

#### Local RFST

The Local RFST involved finding an RF3 amongst rectified RF3 stimuli. For the Local RFST the RF stimuli were matched in global properties (3 corners separated by 120°) and are only distinguishable by local differences in curvature or orientation [see Figure 1B; (28, 48)].

#### RFST stimuli

The base radius of the RF patterns was set at 1° [as in (9)] and the luminance profiles were all Gaussian in cross section (σ = 4′ visual angle). Both patterns in the display (RF3 vs. RF4 or RF3 vs. RRF3) had the same total luminous energy. Weber contrast of 1 was used. The center to center separation of the RF patterns was approximately 3°. The RF's were dispersed on a 7 × 7 grid, creating 49 possible positions for the RF's to appear, which were randomly assigned on each trial. To prevent the patterns from strongly grouping into columns and rows, the patterns were displaced by a random amount (with the range of ±12 arcmin), vertically and horizontally from the grid position.

The stimuli for the RFST were created in MatLab 7.0.4 (Mathworks, Natick, MA, USA), and were presented using a PC (Pentium 4, 3 GHz) driving a Sony Trinitron G520 monitor (100 Hz refresh rate, 1024 × 768 pixels resolution) through the frame buffer of a Cambridge Research Systems (CRS) ViSaGe (CRS, Kent, UK) visual stimulus generator. The background luminance was set to 45 cd/m^2^ and maximum luminance to 90 cd/m^2^, which was calibrated using an Optical OP 200-E photometer (head model number 265). The viewing distance was sustained by the use of a chin rest 65.5 cm from the screen (creating pixels subtending 2′ visual angle). The left and right buttons on a CRS CB6 button box were used to make responses.

#### RFST procedure

A single-interval forced choice reaction time paradigm was used, such that the participant was required to indicate the presence of the target shape via a left (present) or right (absent) button press. The set sizes were randomly interleaved, with the target shape appearing in the display 50% of the time (i.e., half Target Present, half Target Absent). There were 10 practice trials administered for both versions of the RFST to familiarize participants with the task. There were 120 trials per block, with a total of 90 trials per set size (3 blocks × 30 trials p/set size). The blocks of the Global and Local RFST's were interleaved to control for possible practice effects, with the starting block randomized between participants. Reaction time was recorded to 100 μs resolution. Auditory feedback was provided through two different tones, to indicate correct or incorrect responses.

#### The embedded figures test (EFT)

The EFT (24) measures the time (in seconds) taken for an individual to find a simple shape hidden inside a more complex pattern, with a maximum search time of 180 s. The primary measure of performance is the mean response time across the 12 trials (as has been used previously; 9, 25, 24), with the number of completed items (i.e., completed within 180 s) providing a secondary measure of performance.

### Additional tests

Differences in visual acuity, handedness and general cognitive ability can independently influence perceptual organization performance (16, 49, 50), so were included as potential covariates. Visual acuity was assessed using a LogMAR acuity chart (51), where lower scores correspond to poorer visual acuity. Handedness was assessed online using a 10-item version of the Edinburgh Handedness Inventory (http://zhanglab.wikidot.com/handedness), which gives participants a score ranging from −1 (pure left handed) to +1 (pure right handed). A tablet-based version of the digit symbol coding test from the Brief Assessment of Cognition in Schizophrenia (NeuroCog Trials, 2014) provided a brief but reliable estimate of general cognitive ability (52), with participant scores reflecting the number of items completed correctly in 90 s.

### Procedure

The PAb, Social Anhedonia, Cognitive Disorganization, and AQ scales were voluntarily completed in supervised groups in an initial screening session, following a normal laboratory class. Individuals meeting the inclusion criteria on the PAb scale were invited to take part in further testing (total testing time ~2.5 h). The AQ, all four Wisconsin Schizotypy Scales-Brief subscales and the Cognitive Disorganization subscale were re-administered for test-retest reliability purposes. Only selected High and Low PAb participants completed the EFT^4^. For both the RFST and EFT, participants were able to freely move their eyes over the stimuli. Another series of tests followed the RFST, which were part of related experiments (to be reported separately). This research was approved by the Human Research Ethics Committee at the University of Western Australia, and all participants provided informed, written consent. All participation was voluntary and no financial incentives were offered.

### Data analysis

First a data quality check was conducted. Test-retest reliability for the schizotypy scales was as follows: PAb = 0.73; Social Anhedonia = 0.73, Cognitive Disorganization = 0.84 and AQ = 0.77, indicating good or acceptable reliability for all scales. Screening scores were used for group allocations reported below. Next, individuals who scored 3 or more on the Infrequency Scale were excluded from the final sample (*n*_highPAb_ = 2, *n*_lowPAb_ = 2). Outliers (> ± 3 SD) were detected on the digit symbol coding (*n*_lowPAb_ = 3) and age (*n*_lowPAb_ = 6, *n*_highPAb_ = 1), and were removed prior to analysis.

EFT reaction time scores were skewed, hence the logarithm of the reaction time's (recorded in seconds) was taken for the EFT. The anti-log of this data is reported in the Results section below. For the RFST, only correct responses were included, and the lower limit of accuracy was set at 75%. All participants met this criterion for the Global RFST, however, a subset of individuals performed below this criterion on the Local RFST, and were subsequently removed from these analyses (*n*_highPAb_ = 9, *n*_lowPAb_ = 24). There were no differences in accuracy between the High/Low PAb groups (Present and Absent) (see Supplementary Materials for further details, Figure S1).

Next, the median^5^ of the reaction time's for each set size was obtained, and fit with a linear function, [see (9–11)] using Equation (3):

(3) $$\begin{array}{r} Reactiontime=slope\;x\;\left(set\;size\right)+intercept \end{array}$$

The slope of the function represents the rate of increase in reaction time as set size increases and therefore provides a measure of search efficiency. *Slope* was used as the primary indicator of task performance, with steeper search slopes representing reduced search efficiency. The *intercept* provided a secondary outcome measure, and is argued to represent non-search processes, related to signal transmission speed. *R*^2^–a goodness of fit statistic—on average indicated a good fit for the linear functions (equation 3), for both the Global (Target Present = 0.88, Target Absent = 0.92) and Local (Target Present = 0.94, Target Absent = 0.99) RFST's. Present and Absent data were analyzed separately, as noted above (9, 27, 28). In addition, since the Global and Local RFST's are targeting independent processes, they were treated as such in the analyses.

Independent samples *t*-tests were used to explore group differences in slope and intercepts. Effects sizes are represented by Cohen's *d* for the independent samples *t*-tests. Effect sizes are considered small when *d* = 0.20 or approaches that value, medium when *d* = 0.50 or approaching that value, and large when approaching *d* = 0.80 (53). It is expected that the effect sizes will be small-medium, given that medium effect sizes have been found in other PO tasks with clinical samples (2, 18). Analyses were completed in IBM SPSS Statistics Version 22 and GraphPad Prism Version 7^6^.

## Results

### Descriptive statistics

Independent samples *t*-tests (two-tailed) showed that High and Low PAb groups were not significantly different in age, digit symbol coding, handedness or visual acuity. However, compared to the Low PAb groups, the High PAb groups had significantly higher AQ scores and higher levels on *all* schizotypy traits, except Physical Anhedonia (see Table 1). Inspection of the effect sizes (Table 1) and score distributions (Figure S2) for all traits showed that the largest group difference occurred on the PAb scale (as expected), with considerable group overlap on the remaining traits. In addition, the average scores for the High and Low PAb groups for the other schizotypy and AQ traits were within the mid-range of scores (i.e., not below the 50th percentile or above the 90th percentile; see column 2 and 4 in Figure S2).

**Table 1:** Participant characteristics (Mean and SD) in high and low Perceptual Aberration groups.

	**Low PAb (*n* = 146)**	**High PAb (*n* = 83)**	***t***	***p***	***d***
Perceptual aberration	0 (0.00)	5.10 (2.41)	25.62	<0.01^*^	2.99
Magical ideation	1.28 (1.56)	4.23 (2.83)	10.17	<0.01^*^	1.29
Social anhedonia	1.57 (2.27)	3.64 (3.31)	5.58	<0.01^*^	0.73
Physical anhedonia	1.89 (1.91)	2.17 (2.01)	1.04	0.30	0.14
Cognitive disorganization	4.42 (3.15)	7.25 (2.94)	6.03	<0.01^*^	0.93
Total autism quotient	14.10 (6.09)	18.43 (6.81)	4.96	<0.01^*^	0.67
Acuity	−0.11 (0.08)	−0.09 (0.09)	1.42	0.16	0.18
Digit symbol coding	59.20 (8.65)	59.15 (9.25)	−0.03	0.97	−0.01
Age	19.49 (2.81)	19.18 (2.63)	−0.87	0.39	−0.11
Handedness^∧^	0.68 (0.55)	0.70 (0.42)	6931.50	0.12	−1.56

*For the Cognitive Disorganization, data was only available for 176 individuals*.

∧ *Mann-Whitney test used due to negative skew, effect size represented by Z*.

* *p is significant at.05 level (two-tailed)*.

To determine whether covariates should be used in the analyses reported below, correlations were calculated between measures of acuity, handedness, digit symbol coding, and AQ scores, and all measures of PO, i.e., slopes and intercepts on both the Global and Local RFST (for Target Present and Target Absent conditions), and EFT average reaction time scores. Correlations larger than 0.3 were used to identify potential covariates [eg., (54); see Table S2]. No correlations met this criterion. Most traits were weakly and non-significantly correlated with the PO measures, and therefore were not used as covariates. Given prior literature on the association of AQ and PO, the distribution of AQ scores in schizotypy groups was examined. The percentage of High AQ's (≥23) in the High PAb (30.12%) group was higher than the Low PAb (9.59%), presenting difficulty in teasing out the effect of AQ (Figure S3). However, when repeating the analyses after removing these High AQ individuals, the pattern of results remained the same.

## RFST performance

### Global RFST

#### Target present

**Figure 2:**
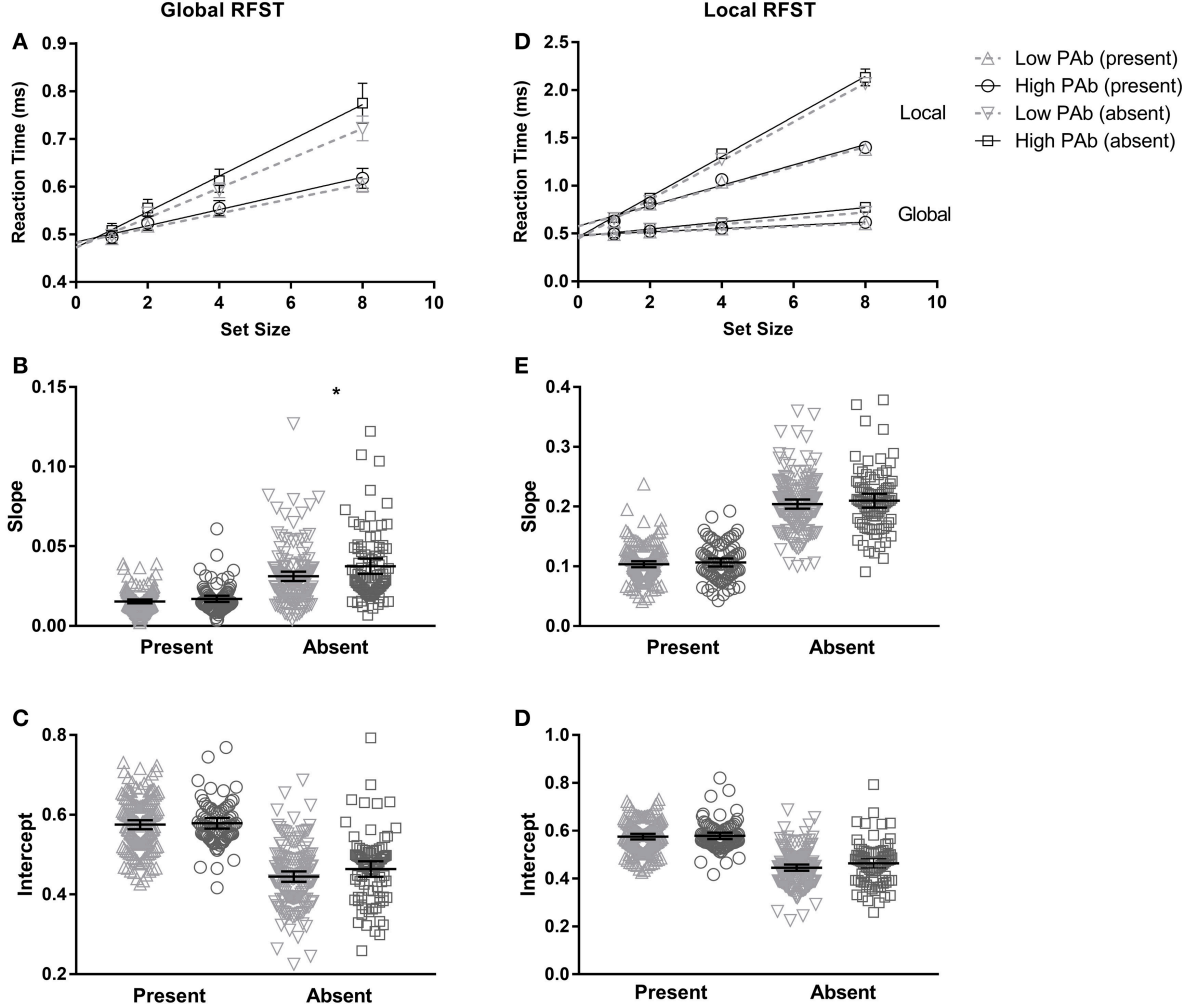
Mean search slopes and intercepts for the High and Low PAb groups for the Global (left panels) and Local (right panels) RFSTs, derived by fitting a linear function when reaction time is plotted against set size (see **A**,**D**). Due to differences in task difficulty between Global and Local, the data are presented on different axes. **(D)** Highlights the difference in task difficulty between Global and Local RFST's, when they are shown on the same axes. Individual slopes **(B,E)** and intercepts **(C,F)** are represented in the bottom panels. The High PAb group exhibited significantly steeper search slopes for the Global Absent RFST only (^*^p is significant at the 0.05 level, two-tailed).

Search slopes tended to be higher for the High PAb group compared to the Low PAb group on the Global Target Present RFST, with a small effect size (Table 2); however, the difference between groups was not statistically significant (Figure 2). No significant difference in processing speed (intercepts) between the High and Low PAb groups was found (small effect size, see Table 2).

**Table 2:** Independent samples *t*-tests comparing High and Low PAb groups on the slopes and intercepts for the linear fits to RFST data.

	***t***	***p***	***d***
**SLOPE**			
Global present	1.519	0.130	0.202
Global absent	2.357	0.019^*^	0.315
Local present	0.680	0.497	0.093
Local absent	0.853	0.408	0.112
**INTERCEPT**			
Global present	0.206	0.837	0.028
Global absent	−0.012	0.991	−0.002
Local present	0.384	0.702	0.054
Local absent	1.664	0.098	0.225

* *p is significant at 0.05 level (two-tailed), df = 227*.

#### Target absent

Significantly steeper search slopes were found on the Global Target Absent RFST, that is, the High PAb group exhibited reduced search efficiency (Figure 2), with a medium effect size^7^. This outcome follows the same pattern of results as the Target Present condition although it was not a significant effect in that case.

### Local RFST

#### Target present

There were no significant differences between High and Low PAb groups on the slopes or intercepts for the Local RFST, and all effect sizes were small.

#### Target absent

There were also no differences between the High and Low PAb groups on Local Target Absent search efficiency, however, the High PAb group exhibited larger intercepts (albeit this was not statistically significant), with small effect sizes (see Table 2 and Figure 2).

### EFT performance

No significant group differences were found on either EFT reaction time scores, *t*_(136)_ = 0.240, *p* = 0.811, *d* = 0.04 (High PAb: *M* = 30.490, *SD* = 1.627, *n* = 49; Low PAb: *M* = 29.803, *SD* = 1.748, *n* = 89), or the number of completed items, *U* = 2046.000, *Z* = −0.654, *p* = 0.513 (High PAb: *M* = 11.102, *SD* = 1.085; Low PAb: *M* = 11.225, *SD* = 0.899). However, a modest significant, positive correlation was observed between EFT reaction time and search efficiency on both the Global (*r* = 0.217, *p* = 0.018, *n* = 118) and Local RFST (*r* = 0.310, *p* = 0.001, *n* = 118), when using Target Present slopes as the measure of performance. In contrast, correlations between the EFT reaction time and Global (*r* = 0.071, *p* = 0.443, *n* = 118) or Local (*r* = 0.180, *p* = 0.051, *n* = 118) Target Absent slopes were non-significant.

## Discussion

This study aimed to explore PO differences in people with High and Low positive (PAb) schizotypal traits using both newer (RFST) and traditional (EFT) measures of aspects of PO. The RFST is similar to the EFT in terms of its visual search properties. However, the RFST offers the significant advantage of allowing us to test whether the PO difficulties observed in high schizotypes may be specifically attributed to differences in global or local processes; in particular, to differences in search efficiency (i.e., rate of change in reaction time as set size increased) or processing speed (including other non-search processes). Contrary to predictions, we found that global processing was significantly *poorer* (rather than enhanced) in the High PAb group relative to the Low PAb group. Specifically, the results showed *lower* search efficiency on the Global RFST in those with High PAb scores, compared to those with Low PAb scores. This difference in global processing had a moderate effect size, but was evident only in the Target Absent condition and not the Target Present condition—which suggests that the underlying mechanism involves differences in decisional processes on the RFST. These findings cannot be attributed to a speed-accuracy trade-off, given that there were no group differences in the proportion of correct responses. In addition, group differences in global processing could not be explained as a result of generalized deficits related to age, digit symbol coding scores, handedness or visual acuity, since High and Low PAb groups did not differ on these variables. Similarly, it is unlikely that group differences in AQ can explain the decreased efficiency in global processing observed, since higher AQ scores have previously been associated with *better* rather than worse performance on the RFST (11). Furthermore, when high AQ scorers were removed from both high and low schizotypy groups, the key results remained unchanged. Importantly, however, the specificity of the difficulty in global processing to *positive* schizotypy alone cannot be assured, since the High PAb group also had high scores on other schizotypal traits. Indeed, prior research shows that schizotypal traits are typically positively correlated (55); hence attempting to isolate a single trait would select a very atypical group not representative of most people with high schizotypy scores.

A notable observation for the remainder of the results is the lack of significant group differences in PO. First, the prediction of a global processing advantage in high schizotypes, as posited by Russell-Smith et al. (25), in terms of speed of processing was not confirmed. High and Low PAb groups did not exhibit any differences in intercept values—indicating that signal transmission speed was similar. Indeed previous evidence indicates that speed of processing is largely intact in high positive schizotypy—which may confer some protection to the onset of schizophrenia [see also (56, 57)]. Second, and significantly, no group differences were observed in local processing abilities on the RFST, in either Target Absent or Target Present conditions; though search efficiency was lower overall on the Local compared to the Global RFST (Figure 2D), suggesting the Local RFST was more difficult, as expected (28, 48). Together with the results outlined above, these findings suggest that high schizotypy is associated with (a) a specific though mild difficulty in global but not local processing, (b) involves poorer search efficiency rather than slower processing speed, (c) cannot be explained as a task difficulty effect, and (d) may arise from altered decision processes when organizing perceptual information into shapes and objects.

Lastly, no significant differences between High and Low PAb groups were found on the EFT task. The unexpected outcome on the EFT appears to be due to close to ceiling accuracy levels in both High and Low PAb groups, which suggests that the EFT is not sufficiently sensitive to differences in PO abilities in this sample. In contrast, the RFST was able to reveal group differences in global processing, that would have previously been missed with the EFT. Indeed, the significant correlation observed between the EFT and RFST [as also found in Almeida et al. 9] suggests the tasks share some common variance, but are not identical. It's important to note that the conceptualisation of local and global is slightly different in the EFT and RFST. The EFT assesses an individuals' ability to disembed local features from a global stimulus, whereas the RFST aimed to isolate the local and global elements. The terms “local” and “global” have taken on a number of different meanings in the literature (33), however, the RFST is one of the few tasks that has attempted to separate local and global components.

The subtle difficulties in global processing captured by the RFST in high-trait schizotypy are consistent with continuum models of psychosis, in demonstrating that anomalies in PO are not limited to schizophrenia but are also observed in healthy individuals at increased risk for psychotic illness (2). However, longitudinal studies tracking global and local processing during development are still needed to determine whether poorer global search efficiency presages the onset of psychotic disorder and conversely, whether intact search efficiency serves as a protective factor. Current models of psychosis risk clearly advocate for a combination of risk factors leading to the development of clinically diagnosable schizophrenia (58–60); though they have not typically included modern measures of PO and lack detail on the precise mechanisms involved. It has previously been suggested that perceptual organization difficulties may, at the phemenological level, translate to individuals having a more fragmented view of the world (61), indicating that intact perceptual organization may protect against the development of a potentially disorganized or anomalous world view (1). The benefit of the current tasks to assess PO lies in providing insight into which specific components of PO may be deficient, setting young people on a trajectory toward more anomalous perceptual experiences and increased risk for psychosis.

A better understanding of global and local processing in schizotypy, i.e., the specific behavioral and neural mechanisms involved, may also provide new avenues for examining well-documented impairments in perceptual organization in schizophrenia (3, 62). For example, the RFST (unlike the EFT) can be easily modified to test the influence of specific stimulus characteristics, such as spatial frequency, which has been argued to be particularly relevant to visual processing deficits in schizophrenia (63, 64) and schizotypy (65). Indeed, further work is needed to determine if poor performance on the RFST is a reliable marker of risk for psychosis and is also observed in patients with schizophrenia. Further work may also consider measuring eye movements [e.g., (66)] as an adjunct measure of global and local processing differences in high and low schizotypy samples. Despite many advantages, alternative tools for assessing PO, such as the Jittered Orientation Visual Integration task, often prove challenging for patients with schizophrenia, with tolerability ratings ranging from low to moderate (67). The RFST offers a quick (~10 min) and easy alternative (~96% average correct responses) that may be refined for use as a screening tool in patients [e.g., (68)].

## Limitations

A number of limitations should be noted. Firstly, this study was restricted to a university student sample, which despite having its advantages, limits the generalizability of these results due to the selective nature of university entrance. Secondly, gender differences in PO should be an important consideration for future research, in light of previous studies which have found that males tend to be faster at RT based tasks (69), and have varied PO difficulties in schizophrenia (70). Thirdly, the interaction between Age, PO, and schizotypy traits was not explored due to the restricted age range of participants. Lastly, in the current study, the small, and often non-significant correlations between negative and disorganized schizotypy traits and RFST measures, suggest that these *other* schizotypy traits have little to no impact on global (or local) processing; though such conclusions need to be tentative. As a whole, the interaction between schizotypy traits and underlying cognitive-perceptual mechanisms is not well understood [e.g., (71)], and should continue to be a focus for future work.

In conclusion, there has been concerted interest in developing reliable and valid tasks for assessing perceptual dysfunction in people with, and at increased risk for, schizophrenia (18, 72). The varieties of RFST available, pave the way for additional, flexible measures of PO that could be used in both clinical and high-risk samples. The RFST is a sensitive assessment tool, and may be particularly useful in helping to better understand the development of visual processing dysfunction prior to illness onset, even when PO processes are relatively preserved (36, 45).

## Author contributions

KP, JB, JD, and DB contributed to the development of the study design. JD and DB created the stimuli and programs for collecting data. KP collected and analyzed the data and produced the manuscript, in partial fulfillment of her Ph.D. JB, JD, and DB provided feedback on the manuscript KP, JB, JD, and DB gave final approval and agreed to be accountable for all aspects of the work.

### Conflict of interest statement

The authors declare that the research was conducted in the absence of any commercial or financial relationships that could be construed as a potential conflict of interest.

## Footnotes

^1^These data was only available for a subset of individuals (*n* = 176).

^2^Equation 1. *R*(θ) = *R*_0_(1+*Asin*(ωθ+ φ)), where ϑ is the angle made with the x-axis, *R*_0_ is the mean radius of the modulated circle, ω is the frequency of modulated cycles in 360^0^ (ω = 3 or 4, denoted RF3 and RF4 respectively) and φ is the angular phase of the shape. Equation 2: The equation for the negatively rectified stimuli [see Local RFST below; (48)] is: $R\left(\theta \right)=R_{0}\left(1+A-|\left(Asin\left(\frac{\omega }{2}\theta +\;\varphi \right)\right)|\right)$.

^3^A recent study found that the targets and distractors of both the Global and Local RFST's were equally salient (28).

^4^Three individuals from the Low PAb group had recently completed the EFT as part of another study, in order to avoid repeat testing—which invalidates responses—scores for these individuals were obtained (with consent), from the earlier study. Data for 4 individuals was missing.

^5^The median was chosen over the mean as there was no single transformation that could normalize the data in all conditions.

^6^The analyses were repeated when removing 9 outliers on slope and 3 outliers on intercept values (Mean ≥ 3 *SD*), however, the outcome did not change.

^7^Following a suggestion from a reviewer, a *post-hoc* exploratory analysis was conducted to determine the impact of age on search efficiency. Groups were broken down as follows: 17–19 (High PAb: 67.5%, Low PAb: 70.5%), 20-24 (High PAb: 25.3%, Low PAb: 21.9%), 25–31 (High PAb: 7.2%, Low PAb: 7.5%). An interaction of Age × PAb group was found, such that High PAb group showed significantly steeper search slopes compared to the Low PAb for the Youngest (17–19) group *only*. However, given the small proportion of the sample in the older age groups, it is difficult to interpret the overall effect of age on search efficiency.

## Abbreviations

EFT: Embedded Figures Test
RFST: Radial Frequency Search Task
PAb: Perceptual Aberration
PO: Perceptual Organization
RF: Radial Frequency
AQ: Autism Quotient.

## References

[1] de-Wit L Wagemans J Individual Differences in Local and Global Perceptual Organization. Oxford: Oxford University Press (2015).

[2] Panton KR Badcock DR Badcock JC. A metaanalysis of perceptual organization in schizophrenia, schizotypy, and other high-risk groups based on variants of the Embedded Figures Task. Front Psychol. (2016) 7:237. 10.3389/fpsyg.2016.00237 26941688 PMC4763090

[3] Silverstein SM. Visual perception disturbances in schizophrenia: a unified model. In: Li M Spaulding WD editors. The Neuropsychopathology of Schizophrenia. Switzerland: Springer (2016). p. 77–132. 10.1007/978-3-319-30596-7_4 27627825

[4] Silverstein SM Keane BP. Perceptual organization impairment in schizophrenia and associated brain mechanisms: review of research from 2005 to 2010. Schizophr Bull. (2011) 37:690–9. 10.1093/schbul/sbr052 21700589 PMC3122298

[5] Palmer SE Vision Science: Photons to Phenomenology. Cambridge, MA: MIT Press (1999).

[6] Wagemans JJH Elder JH Kubovy M Palmer SE Peterson MA Singh M A century of gestalt psychology in visual perception: I. perceptual grouping and figure–ground organization. Psychol Bull. (2012) 138:1172–217. 10.1037/a0029333 22845751 PMC3482144

[7] Carter O Bennett D Nash T Arnold S Brown L Cai RY Sensory integration deficits support a dimensional view of psychosis and are not limited to schizophrenia. Transl Psychiatry (2017) 7:e1118. 10.1038/tp.2017.69 PMC5534945 28485725

[8] Uhlhaas PJ Silverstein SM. Perceptual organization in schizophrenia spectrum disorders: empirical research and theoretical implications. Psychol Bull. (2005) 131:618–32. 10.1037/0033-2909.131.4.618 16060805

[9] Almeida RA Dickinson JE Maybery M Badcock JC Badcock DR. A new step towards understanding Embedded Figures Test performance in the autism spectrum: the radial frequency search task. Neuropsychologia (2010) 48:374–81. 10.1016/j.neuropsychologia.2009.09.024 19786040

[10] Almeida RA Dickinson JE Maybery M Badcock JC Badcock DR. Visual search performance in the autism spectrum II: the radial frequency search task with additional segmentation cues. Neuropsychologia (2010) 48:4117–24. 10.1016/j.neuropsychologia.2010.10.009 20946906

[11] Almeida RA Dickinson JE Maybery MT Badcock JC Badcock DR. Visual search targeting either local or global perceptual processes differs as a function of autistic-like traits in the typically developing population. J Autism Dev Disord. (2013) 42:1272–86. 10.1007/s10803-012-1669-7 23054202

[12] Almeida RA Dickinson JE Maybery MT Badcock JC Badcock DR. Enhanced global integration of closed contours in individuals with high levels of autistic-like traits. Vision Res. (2014) 103:109–15. 10.1016/j.visres.2014.08.015 25175114

[13] Dakin S Frith U. Vagaries of visual perception in autism. Neuron (2005) 48:497–507. 10.1016/j.neuron.2005.10.018 16269366

[14] Butler PD Abeles IY Silverstein SM Dias EC Weiskopf NG Calderone DC. An event-related potential examination of contour integration deficits in schizophrenia. Front Psychol. (2013) 4:132. 10.3389/fpsyg.2013.00132 23519476 PMC3604636

[15] Feigenson KA Keane BP Roché MW Silverstein SM Contour integration impairment in schizophrenia and first episode psychosis: State or trait? Schizophr Res. (2014) 159:515–20. 10.1016/j.schres.2014.09.028 25306205 PMC4254521

[16] Keane BP Kastner S Paterno D Silverstein SM. Is 20/20 vision good enough? Visual acuity differences within the normal range predict contour element detection and integration. Psychon Bull Rev. (2014) 22:121–7. 10.3758/s13423-014-0647-9 24845876 PMC4240750

[17] Silverstein SM Harms MP Carter CS Gold JM Keane BP MacDonald AIII. Cortical contributions to impaired contour integration in schizophrenia. Neuropsychologia (2015) 75:469–80. 10.1016/j.neuropsychologia.2015.07.003 26160288 PMC4546547

[18] Silverstein SM Keane BP Barch DM Carter CS Gold JM Kovács I. Optimization and validation of a visual integration test for schizophrenia research. Schizophr Bull. (2012) 38:125–34. 10.1093/schbul/sbr141 22021658 PMC3245579

[19] Keane BP Paterno D Kastner S Silverstein SM. Visual integration dysfunction in schizophrenia arises by the first psychotic episode and worsens with illness duration. J Abnorm Psychol. (2016) 125:543–9. 10.1037/abn0000157 27030995 PMC4850085

[20] Gooding DC Iacono WG Schizophrenia through the lens of a developmental psychopathology perspective. In: Cicchetti D Cohen DJ editors. Manual of Developmental Psychopathology, Vol. 2. Risk, Disorder, and Adaptation. New York, NY: Wiley (1995).

[21] Jablensky A McNeil TF Morgan VA. Barbara Fish and a short history of the neurodevelopmental hypothesis of schizophrenia. Schizophr Bull. (2017) 43:1158–63. 10.1093/schbul/sbx094 29036635 PMC5737550

[22] Weinberger DR. Implications of normal brain development for the pathogenesis of schizophrenia. Arch Gen Psychiatry (1987) 44:660–9. 10.1001/archpsyc.1987.01800190080012 3606332

[23] Debbané M Eliez S Badoud D Conus P Flückiger R Schultze-Lutter F. Developing psychosis and its risk states through the lens of schizotypy. Schizophr Bull. (2015) 41, S396–407. 10.1093/schbul/sbu176 25548386 PMC4373628

[24] Witkin HA Oltman PK Raskin E Karp SS A Manual for the Embedded Figures Test. Palo Alto, CA: Consulting Psychologists Press (1971).

[25] Russell-Smith S Maybery MT Bayliss D. Are the autism and positive schizotypy spectra diametrically opposed in local versus global processing? J Autism Dev Disord. (2010) 40:968–77. 10.1007/s10803-010-0945-7 20108115

[26] Wilkinson F Wilson HR Habak C. Detection and recognition of radial frequency patterns. Vision Res. (1998) 38:3555–68. 10.1016/S0042-6989(98)00039-X 9893789

[27] Wolfe JM. Guided search 2.0 a revised model of visual search. Psychon Bull Rev. (1994) 1:202–38. 10.3758/BF03200774 24203471

[28] Dickinson JE Haley K Bowden VK Badcock DR. Visual search reveals a critical component to shape. J Vis. (2018) 18:2. 10.1167/18.2.2 29392277

[29] Bell J Badcock DR. Narrow-band radial frequency shape channels revealed by sub-threshold summation. Vision Res. (2009) 49:843–50. 10.1016/j.visres.2009.03.001 19275912

[30] Bowden VK Dickinson JE Fox AM Badcock DR. Global shape processing: a behavioral and electrophysiological analysis of both contour and texture processing. J Vis. (2015) 15:18. 10.1167/15.13.18 26401625

[31] Dickinson JE Bell J Badcock DR. Near their thresholds for detection, shapes are discriminated by the angular separation of their corners. PLoS ONE (2013) 8:e66015. 10.1371/journal.pone.0066015 23741521 PMC3669261

[32] Wilkinson F James TW Wilson HR Gati JS Menon RS Goodale MA. An fMRI study of the selective activation of human extrastriate form vision areas by radial and concentric gratings. Curr Biol. (2000) 10:1455–8. 10.1016/S0960-9822(00)00800-9 11102809

[33] Milne L Szczerbinski M. Global and local perceptual style, field-independence, and central coherence: an attempt at concept validation. Adv Cogn Psychol. (2009) 5:1–26. 10.2478/v10053-008-0062-8 20523847 PMC2864999

[34] Winterstein BP Silvia PJ Kwapil TR Kaufman JC Reiter-Palmon R Wigert B Brief assessment of schizotypy: Developing short forms of the Wisconsin Schizotypy Scales. Pers Individ Dif. (2011) 51:920–4. 10.1016/j.paid.2011.07.027

[35] Schuldberg D London A. Psychological differentiation and schizotypal traits: negative results with the Group Embedded Figures Test. Percept Mot Skills (1989) 68:1219–26. 10.2466/pms.1989.68.3c.1219 2762087

[36] Ettinger U Mohr C Gooding DC Cohen AS Rapp A Haenschel C. Cognition and brain function in schizotypy: a selective review. Schizophr Bull. (2015) 41, S417–26. 10.1093/schbul/sbu190 25810056 PMC4373634

[37] Gross GM Silvia PJ Barrantes-Vidal N Kwapil TR. The dimensional structure of short forms of the Wisonsin Schizotypy Scales. Schizophr Res. (2015) 166:80–5. 10.1016/j.schres.2015.05.016 26036815

[38] Mason O Linney Y Claridge G. Short scales for measuring. Schizophr Res. (2005) 78:293–96. 10.1016/j.schres.2005.06.020 16054803

[39] Chapman LJ Chapman JP Infrequency Scale. Unpublished Test. Greensboro, NC: Department of Psychology, University of North Carolina at Greensboro (1983).

[40] Jablensky A McGrath J Herman H Castle D Gureje O Evans M. Psychotic disorders in urban areas: an overview of the study on low prevalence disorders. Aust N Z J Psychiatry (2000) 34:221–36. 10.1080/j.1440-1614.2000.00728.x 10789527

[41] Castle D Jablensky A McGrath J Carr V Morgan V Waterreus A. The diagnostic interview for psychoses (DIP): development, reliability and applications. Psychol Med. (2006) 36:69–80. 10.1017/S0033291705005969 16194284

[42] Fonseca-Pedrero E Ortuño-Sierra J Mason OJ Muñiz J The oxford–liverpool inventory of feelings and experiences short version: further validation. Pers Individ Dif. (2015) 86:338–43. 10.1016/j.paid.2015.06.041

[43] Fonseca-Pedrero E Palno M Ortuño-Sierra J Lemos-Giráldez S Muñiz J. Dimensionality of the Wisconsin Schizotypy scales-brief forms in college students. Sci World J. (2013) 2013:1–8. 10.1155/2013/625247 24319377 PMC3844248

[44] Baron-Cohen S Wheelwright S Skinner R Martin J Clubley E. The autism-spectrum quotient (AQ): evidence from asperger syndrome/high-functioning autism, males and females, scientists and mathematicians. J Autism Dev Disord. (2001) 31:5–17. 10.1023/A:1005653411471 11439754

[45] Cribb SJ Badcock JC Maybery MT Badcock DR. Dissociation of local and global contributions to detection of shape with age. J Exp Psychol. (2016) 42:1761–9. 10.1037/xhp0000257 27379873

[46] Dickinson JE McGinty J Webster KE Badcock DR. Further evidence that local cues to shape in RF patterns are integrated globally. J Vis. (2012) 12:1–17. 10.1167/12.12.16 23197768

[47] Loffler G Wilson HR Wilkinson F. Local and global contributions to shape discrimination. Vision Res. (2003) 43:519–30. 10.1016/S0042-6989(02)00686-7 12594998

[48] Dickinson JE Cribb SJ Riddell HR Badcock DR. Tolerance for local and global differences in the integration of shape information. J Vis. (2015) 15:1–24. 10.1167/15.3.21 25814547

[49] Powell JL Kemp GJ García-Finaña M. Association between language and spatial laterality and cognitive ability: an fMRI study. Neuroimage (2012) 59:1818–29. 10.1016/j.neuroimage.2011.08.040 21889594

[50] Sheppard LD Vernon PA Intelligence and speed of information-processing: a review of 50 years of research. Pers Individ Dif. (2008) 44:535–51. 10.1016/j.paid.2007.09.015

[51] Oduntan OA Mashige KP Raliavhegwa-Makhado M A comparison of two methods of logMAR visual acuity data scoring for statistical analysis. South Afr Optomet. (2009) 63:155–63. 10.4102/aveh.v68i3.162

[52] Atkins AS Davis VG Tseng T Vaughan A Harvey PB Narasimhan M Validation of the Tablet-based Brief Assessment of Cognition (BAC App) for Schizophrenia (2014). Available online at: http://www.neurocogtrials.com/10.1016/j.schres.2016.10.010 27771201

[53] Cohen J Statistical Power Analysis for the Behavioral Sciences. New York, NY: Academic Press (1977).

[54] Crawford JR Garthwaite PH Ryan K. Comparing a single case to a control sample: testing for neuropsychological deficits and dissociations in the presence of covariates. Cortex (2011) 47:1166–78. 10.1016/j.cortex.2011.02.017 21458788

[55] Gross GM Silvia PJ Barrantes-Vidal N Kwapil TR. Psychometric properties and validity of short forms of the Wisconsin Schizotypy Scales in two large samples. Schizophr Res. (2012) 134:267–72. 10.1016/j.schres.2011.11.032 22189258

[56] Badcock JC Clark ML Pedruzzi RA Morgan VA Jablensky A. Intact speed of processing in a community-based sample of adults with high schizotypy: a marker of reduced psychosis risk? Psychiatry Res. (2015) 228:531–7. 10.1016/j.psychres.2015.06.003 26117248

[57] Chun CA Minor KS Cohen AS Neurocognition in psychometrically defined college schizotypy samples: we are not measuring the “right stuff”. J Internat Neuropsychol Soc. (2013) 19:324–37. 10.1017/S135561771200152X 23448879

[58] de Leede-Smith S Roodenrys S Horsley L Matrini S Mison E Barkus E Neurological soft signs: Effects of trait schizotypy, psychological distress and auditory hallucination predisposition. Schizophr Res Cogn. (2017) 7:1–7. 10.1016/j.scog.2016.11.001 28740822 PMC5514310

[59] Fusar-Poli P Bonoldi I Yung AR Borgwardt S Kempton MJ Valmaggia L. Predicting psychosis: meta-analysis of transition outcomes in individuals at high clinical risk. Arch Gen Psychiatry (2012) 69:220–9. 10.1001/archgenpsychiatry.2011.1472 22393215

[60] Murray RM Bhavsar V Tripoli G Howes O. 30 years on: how the neurodevelopmental hypothesis of schizophrenia morphed into the developmental risk factor model of psychosis. Schizophr Bull. (2017) 43:1190–6. 10.1093/schbul/sbx121 28981842 PMC5737804

[61] Uhlhaas PJ Mishara AL. Perceptual anomolies in schizophrenia: integrating phenomology and cognitive neuroscience. Schizophr Bull. (2007) 33:142156. 10.1093/schbul/sbl047 17118973 PMC2632288

[62] Phillips W Clark A Silverstein SM. On the functions, mechanisms, and malfunctions of intracortical contextual modulation. Neurosci Biobehav Rev. (2015) 52:1–20. 10.1016/j.neubiorev.2015.02.010 25721105

[63] Butler PD Javitt DC. Early-stage visual processing deficits in schizophrenia. Curr Opin Psychiatry (2005) 18:151–7. 10.1097/00001504-200503000-00008 16639168 PMC1994776

[64] Laycock R Crewther SG Crewther DP. A role for the ‘magnocellular advantage’ in visual impairments in neurodevelopmental and psychiatric disorders. Neurosci Biobehav Rev. (2007) 31:363–76. 10.1016/j.neubiorev.2006.10.003 17141311

[65] Cappe C Herzog MH Herzig DA Brand A Mohr C. Cognitive disorganisation in schizotypy is associated with deterioration in visual backward masking. Psychiatry Res. (2012) 200:652–9. 10.1016/j.psychres.2012.07.001 22921599

[66] Levy DL Sereno AB Gooding DC O'Driscoll GA. Eye tracking dysfunction in schizophrenia: characterization and pathophysiology. In: Swerdlow N Editor. Behavioral Neurobiology of Schizophrenia and Its Treatment. Berlin: Springer (2010). p. 311–47. 10.1007/7854_2010_60 PMC3212396 21312405

[67] Strauss ME McLouth CJ Barch DM Carter CS Gold JM Luck SJ. Temporal stability and moderating effects of age and sex on CNTRaCS task performance. Schizophr Bull. (2013) 40:835–44. 10.1093/schbul/sbt089 23817024 PMC4059430

[68] Karageorgiou E Schulz SC Gollub RL Andreasen NC Ho BC Lauriello J. Neuropsychological testing and structural magnetic resonance imaging as diagnostic biomarkers early in the course of schizophrenia and related psychoses. Neuroinformatics (2011) 9:321–33. 10.1007/s12021-010-9094-6 21246418 PMC3116989

[69] Roivainen E Gender differences in processing speed: a review of recent research. Learn Individ Differ. (2011) 21:145–9. 10.1016/j.lindif.2010.11.021

[70] Joseph J Bae G Silverstein SM. Sex, symptom, and premorbid social functioning associated with perceptual organization dysfunction in schizophrenia. Front Psychol. (2013) 4:547. 10.3389/fpsyg.2013.00547 23986732 PMC3753434

[71] Debbané M Badoud D Balanzin D Eliez S. Broadly defined risk mental states during adolescence: disorganization mediates positive schizotypal expression. Schizophr Res. (2013) 147:153–6. 10.1016/j.schres.2013.03.012 23570898

[72] Green MF Butler PD Chen Y Geyer MA Silverstein S Wynn JK. Perception measurement in clinical trials of schizophrenia: promising paradigms from CNTRICS. Schizophr Bull. (2009) 35:163–81. 10.1093/schbul/sbn156 19023123 PMC2635893

