# Supplementary material for: Poorer Search Efficiency in Healthy Young Adults With High Schizotypal Personality Traits
Source: Front Psychiatry. 2018 Jul 12;9:285. doi: 10.3389/fpsyt.2018.00285 (PMC6052133; doi:10.3389/fpsyt.2018.00285)
Supplement: Supplementary file 1 [file Data_Sheet_1.pdf]

Supplementary Material

Poorer search efficiency in healthy young adults with high schizotypal personality traits

Kirsten R. Panton[1*], Johanna C. Badcock[2, 3], J. Edwin Dickinson[1] and David R. Badcock[1]

*Corresponding Author:
Kirsten Panton
School of Psychological Science
University of Western Australia
35 Stirling Highway, Crawley
Western Australia, 6009, Australia
Tel: (+61 8) 6488 3281
Email: kirsten.panton@research.uwa.edu.au

Supplementary results

To determine whether there were group differences in accuracy between the Global and Local RFST's, four separate independent samples *t*-tests were conducted. No differences between High and Low PAb groups was found on Global Present ($t(227) = -0.111$, $p = .912$, $d = -0.030$), Global Absent ($t(227) = 0.129$, $p = 898$, $d = -0.111$), Local Present ($t(227) = -0.221$, $p = .825$, $d = -0.016$) or Local Absent ($t(227) = -0.756$, $p = .450$, $d = 0.019$) conditions, suggesting there was no group difference in accuracy.

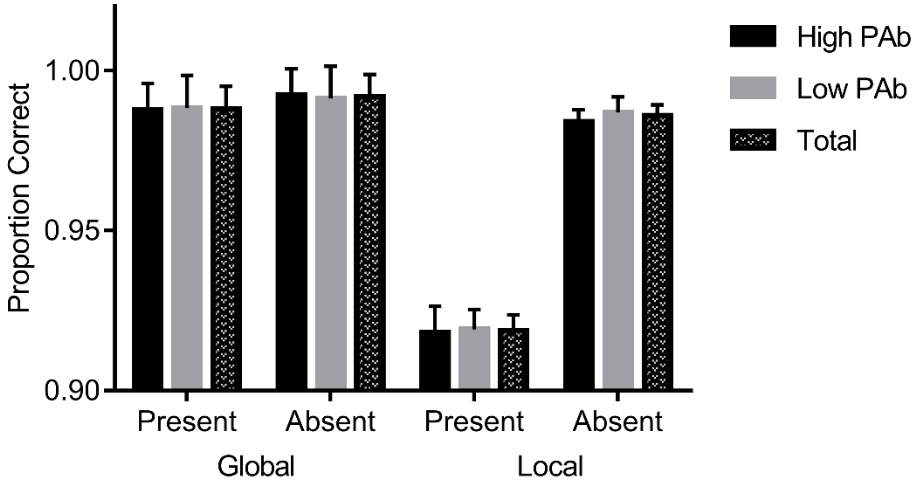

Figure S1. The proportion of correct responses for the High and Low PAb groups for the Global and Local RFST's (Present and Absent).

Table S1. Means and standard deviations for schizotypy samples (all university/college samples) across different studies.

	N	*M* (*PAb*)	*SD* (*PAb*)	*Range* (*PAb*)	*% Males*	*M* (*Age*)
Entire screening sample	2693	.95	1.98	0-15	34.6*	19.62
Winterstein et al. (2011)	1144	1.32	2.27	0-15	24.0	22.90
Fonseca-Pedrero et al. (2013)	1349	1.12	1.88	0-13	21.1	20.48
Gross et al. (2015)	2292	1.29	2.12	0-15	24.0	19.50

**Note:* 3.5% of the sample did not specify a gender.

Table S2. Correlations between schizotypy traits and participant characteristics with measures of PO.

	Global Slope Present	Global Slope Absent	Local Slope Present	Local Slope Absent	Global Intercept Present	Global Intercept Absent	Local Intercept Present	Local Intercept Absent
Schizotypy traits								
Magical Ideation	.144*	.116	.106	.128	030	.118	.048	.056
Social Anhedonia	.069	-.038	-.035	-.151*	-.097	-.115	-.144*	-.013
Physical Anhedonia	-.003	.052	-.021	.009	-.114	-.122	.016	-.036
Cognitive Disorganisation^	.041	.045	-.089	.011	.098	.117	.108	-.002
Total Autism Quotient	.027	.048	-.085	-.067	.036	.057	.086	.053
Digit Symbol Coding^	-.168*	-.080	-.050	.030	-.155*	-.248*	-.142*	-.217*
Acuity^	.016	-.034	.010	.127	.178*	.188*	.090	.006
Age	.101	.156*	-.004	.013	.044	-.077	.072	.027

***Note:* *p is significant at .05 level (two-tailed); ^ Pearson's correlations.

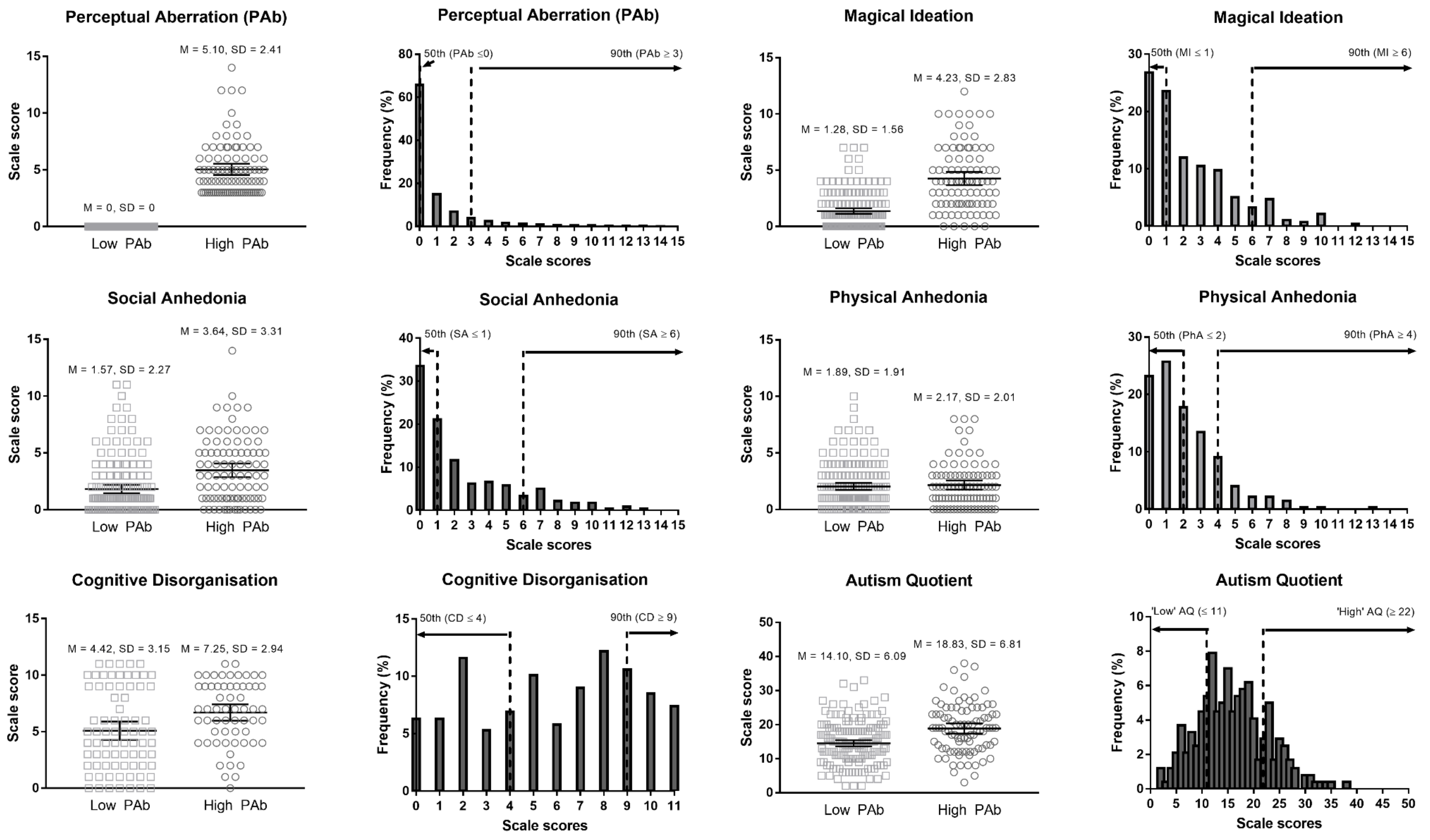

Figure S2. Distribution of schizotypy and AQ scores between High and Low PAb groups are presented in column 1 and 3. Frequency distribution of these traits using re-test scores (apart from the PAb, where screening scores were used) are presented in column 2 and 4. The dotted lines on columns 2 and 4 represent the 50th and 90th percentile cut-offs (aside from the AQ, where a cut-off from a previous study was used, Almeida et al. (2010a). *Note:* error bars represent 95% CI.

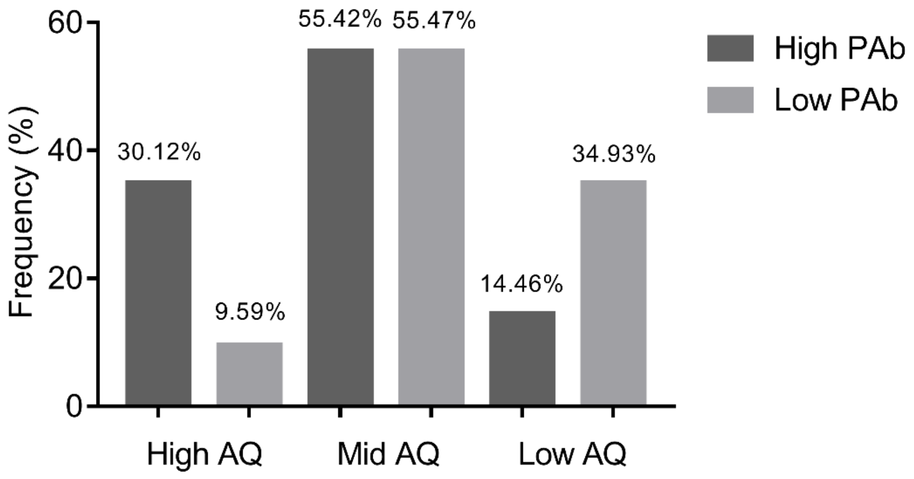

Figure S3. Distribution of High (≥23) and Low (≤11) AQ scores between the High and Low PAb groups.

References

Almeida, R. A., J. E. Dickinson, M. Maybery, J. C. Badcock, and D. R. Badcock. 2010a. "A new step towards understanding Embedded Figures Test performance in the autism spectrum: the radial frequency search task." *Neuropsychologia* 48:374-381. doi: 10.1016/j.neuropsychologia.2009.09.024.

Fonseca-Pedrero, E., M. Palno, J. Ortuño-Sierra, S. Lemos-Giráldez, and J. Muñiz. 2013. "Dimensionality of the Wisconsin Schizotypy Scales-Brief Forms in College Students." *The Scientific World Journal* 2013:1-8. doi: http://dx.doi.org/10.1155/2013/625247.

Gross, G. M., P. J. Silvia, N. Barrantes-Vidal, and T. R. Kwapil. 2015. "The dimensional structure of short forms of the Wisonsin Schizotypy Scales." *Schizophrenia Research* 166 (1):80-85. doi: http://dx.doi.org/10.1016/j.schres.2015.05.016.

Winterstein, B. P., P. J. Silvia, T. R. Kwapil, J. C. Kaufman, R. Reiter-Palmon, and B. Wigert. 2011. "Brief assessment of schizotypy: Developing short forms of the Wisconsin Schizotypy Scales. ." *Personality and Individual Differences* 51:920-924. doi: 10.1007/s10862-011-9242-9.
